# Development of 18 microsatellite markers for *Salvia pratensis*

**DOI:** 10.1002/aps3.11316

**Published:** 2020-01-22

**Authors:** Karol Krak, Petr Vít, Jan Douda, Bohumil Mandák

**Affiliations:** ^1^ Faculty of Environmental Sciences Czech University of Life Sciences in Prague Kamýcká 129 CZ‐16521 Czech Republic; ^2^ Institute of Botany of the Czech Academy of Sciences Zámek 1 CZ‐252 43 Průhonice Czech Republic

**Keywords:** Lamiaceae, microsatellites, phylogeography, *Salvia pratensis*

## Abstract

**Premise:**

Microsatellite markers were developed for the perennial herb *Salvia pratensis* (Lamiaceae), a species representative of European dry grasslands. The development of microsatellite markers is needed for genetic and phylogeographical studies of species from the genus *Salvia*.

**Methods and Results:**

We used low‐coverage Illumina sequencing to identify microsatellite loci. Based on these data, we have developed 18 polymorphic microsatellite markers with the number of alleles per locus ranging from two to 15. The levels of observed and expected heterozygosity ranged from 0.05 to 0.95 and from 0.05 to 0.89, respectively. The majority of the markers successfully cross‐amplified in other *Salvia* species.

**Conclusions:**

The markers were shown to be suitable for population genetic and phylogeographic studies in *S. pratensis* as well as in related species (*S. aethiopis*,* S. austriaca*,* S. glutinosa*,* S. nemorosa*,* S. nutans*, and *S. verticillata*) and will be used in the broader context to trace the origins of European dry grasslands.

*Salvia pratensis* L. (Lamiaceae) is a perennial herb distributed throughout Europe from the Volga River in the east, to northern Spain in the west, to the southern European peninsulas (Balkan and Apennine) in the south, and to the Netherlands, Poland, and Germany in the north (Kaplan et al., 2018). A few locations are also recorded in the British Isles, where the species is protected (Kaplan et al., 2018). The species is diploid with 2*n* = 2*x* = 18 chromosomes (e.g., Van Loon, 1980). It is a typical representative of European temperate dry grassland vegetation. Grasslands represent one of the most species‐rich but threatened ecosystems in temperate Europe (Habel et al., 2013). Understanding the phylogeographic history of such species is crucial for determining the postglacial history of these endangered ecosystems and is expected to facilitate the development of efficient conservation strategies.

A total of 29 microsatellite markers have already been developed for *S. officinalis* L. (Molecular Ecology Resources Primer Development Consortium et al., 2010; Radosavljević et al., 2011, 2012), a species representing a sister group to *S. pratensis* (Will and Claßen‐Bockhoff, 2017). Twenty of these loci were tested for cross‐amplification to *S. pratensis*, but successful cross‐amplification has been reported for only five (Radosavljević et al., 2011). For a state‐of‐the‐art computational approach, this low number of microsatellite markers is inadequate for a comprehensive phylogeographical or population genetic study. Therefore, we set out to develop a set of markers specifically for *S. pratensis*. This marker set was tested for cross‐amplification in other species of *Salvia* L. (*S. aethiopis* L., *S. austriaca* Jacq., *S. glutinosa* L., *S. nemorosa* L., *S. nutans* L., and *S. verticillata* L.).

## METHODS AND RESULTS

We used low‐coverage Illumina sequencing to identify microsatellite loci for *S. pratensis*. Total genomic DNA was extracted from one individual using the DNeasy Plant Mini Kit (QIAGEN, Hilden, Germany) and a sequencing library was prepared using the NEBNext Ultra DNA Library Prep Kit (New England Biolabs, Ipswich, Massachusetts, USA), as described in Belyayev et al. (2019). Paired‐end sequencing with 2 × 300‐bp reads was carried out using the Illumina MiSeq platform (Macrogen, Seoul, South Korea). The *S. pratensis* library was sequenced together with nine other libraries.

The sequencing resulted in 812,212 read pairs. The raw reads were trimmed using Trimmomatic version 0.36 (Bolger et al., 2014) to remove adapters and low‐quality bases using the following settings: ILLUMINACLIP 2:30:10, LEADING:20, TRAILING:20 SLIDINGWINDOW:4:20, and MINLEN:85. The trimmed reads were mapped on the chloroplast genome of *S. miltiorrhiza* Bunge (Qian et al., 2013) to remove reads of cpDNA origin using Bowtie 2 (Langmead and Salzberg, 2012) with default settings. The unmapped reads were used for microsatellite locus filtering using SSR_pipeline 0.951 (Miller et al., 2013) with the following microsatellite search parameter settings: minimum flanking region length 40 bp; minimum number of repeats seven, six, and five for di‐, tri‐, and tetranucleotides, respectively. Primers were designed for the reads containing microsatellites by running additional microsatellite filtering using MSATCOMMANDER 0.8.2 (Faircloth, 2008), using the same microsatellite search settings as in SSR_pipeline and the ‘design primers with M13 tag’ option turned on. This approach yielded 2356 candidate microsatellite loci.

Altogether, 78 candidate loci containing perfect repeat motifs were tested for amplification using seven individuals from four distinct populations (for the origin of populations, see Appendix 1). The primer tailing method, as described in Schuelke (2000), was used to amplify and fluorescently label the resulting PCR products. The PCRs were carried out in 5‐μL reactions containing 1× concentration of QIAGEN Multiplex PCR Mix (QIAGEN), 0.05 μM of the forward primer, 0.2 μM of both the reverse and M13 primers, and 10 ng of template DNA. The cycling conditions were as follows: initial denaturation at 95°C for 15 min followed by 25 cycles of denaturation at 95°C for 30 s, annealing at 55°C for 30 s, and elongation at 72°C for 2 min, complemented by an additional 10 cycles with the annealing temperature decreased to 50°C, and a final elongation step at 72°C for 10 min. The PCR products were first checked by agarose gel electrophoresis. Markers yielding clear non‐ladder‐like banding patterns within the range of 100–500 bp in at least six out of the seven individuals analyzed were further examined on the ABI 3500 automatic sequencer (Applied Biosystems, Foster City, California, USA). For this, 1 μL of non‐diluted PCR products was mixed with 12 μL of Hi‐Di Formamide (Applied Biosystems) and 0.1 μL of GeneTrace 500 LIZ (Carolina Biosystems, Prague, Czech Republic) internal size standard prior to the fragment analysis. Twenty‐six markers showing distinct and easily readable fragments with no more than two alleles per individual were considered for further experiments. Variability was quantified by amplifying the suitable candidate loci using additional plant material from the above‐mentioned four populations (each population represented by 19–20 individuals). During these tests, eight markers were shown to amplify more than two alleles in at least some of the individuals, indicating potential duplication of these microsatellite loci, and were therefore discarded. The remaining 18 markers were all polymorphic (Table 1). The allele sizes were determined using the software GeneMarker 2.6.4 (SoftGenetics, State College, Pennsylvania, USA). The number of alleles, levels of observed and expected heterozygosity, inbreeding coefficient, and deviation from Hardy–Weinberg equilibrium, using Fisher's exact test, were estimated using the R package diveRsity (Keenan et al., 2013) for each locus and population (Table 2). The number of alleles per locus and population varied from two to 15. The levels of observed and expected heterozygosity per locus and per population ranged from 0.05 to 0.95 and from 0.05 to 0.89, respectively. For all loci, except SP_Di_24, SP_Tri_04, and SP_Tri_06, significant deviation (*P <* 0.05) from Hardy–Weinberg equilibrium was observed in at least one of the populations tested. However, only one locus (SP_Tri_17) deviated in all four populations.

**Table 1:** Characteristics of the 18 polymorphic microsatellite loci developed for *Salvia pratensis*.

Locus^a^	Primer sequences (5′–3′)	Repeat motif	Allele size range (bp)	GenBank accession no.
SP_Tri_36	F: ACTCCTCTCCGGCATAGTTTC	(GTT)_9_	160–202	MN219563
	R: CGTCTTGTGTGGCGCAG			
SP_Di_24	F: CCAGCATTTGACGCAATGG	(AG)_8_	209–221	MN219552
	R: GGACCTGCAAACCAAGAACG			
SP_Di_25^b^	F: TCCCATGCAGGTTATCCACC	(GT)_14_	302–337	MN219553
	R: ACAAGCAGCAGCCTCTTAC			
SP_Di_31	F: TGTGAGTGTTGACTTCTTCAATCG	(AG)_8_	282–332	MN219549
	R: GAATGCAATGGCCGGCAG			
SP_Di_27^b^	F: TCCTCGTTCAGCAACTGCC	(CT)_7_	247–352	MN219554
	R: AGGCGCAATTCGACCATTC			
SP_Tri_34	F: TTCCTCTCCTCAAATTCAAACC	(AGC)_6_	223–259	MN219562
	R: AGGAAGATCCAGATCGCCAC			
SP_Di_30	F: CCTTAGCCATCCTCTGATCC	(GT)_11_	156–204	MN219556
	R: CAGCCCACCTAGCAAATACC			
SP_Di_02	F: TTCCAGAAATCCTAAATGCCAG	(AG)_7_	174–214	MN219548
	R: TTCCAGAAATCCTAAATGCCAG			
SP_Di_06^c^	F: CGTTTGGGCAGCTTTCAAC	(AT)_7_	219–236	MN219551
	R: GTCATCAAGAAGCACTGACCC			
SP_Di_04^b^	F: ATTCGTTTGTACGCGACGG	(AT)_7_	231–260	MN219550
	R: AGGGATCTAAGTAGAATCACCAAATG			
SP_Di_08	F: TGGCGGAGGTATTGTTGCC	(CT)_7_	162–208	MN219555
	R: TCTGCTGCCGCGTATTCAC			
SP_Tri_17	F: TGTGCGTTCGTGTCAACAG	(AAT)_6_	202–262	MN219561
	R: CGAAGAAGCTCGTCTCTACAAAG			
SP_Tri_23	F: CAAACGGTGTGGAACCGAG	(CTT)_8_	230–245	MN219564
	R: CTGGCAGTGGCAAGAAATTAAG			
SP_Tri_04^b^	F: CCATTTATTCTCTCAGGTATGCCC	(ACC)_6_	196–207	MN219557
	R: TCAGTATCCTCTTACCATTGCTTC			
SP_Te_07^b^	F: ATATAAACGGTGCTCACAGAAG	(ATGT)_9_	165–215	MN219547
	R: TTCGAGTTCACCACGAGGG			
SP_Tri_06	F: CTCTGAGCTCCCGCAACC	(AAT)_6_	165–195	MN219558
	R: TTATTGCTGTCACGTGTCCC			
SP_Tri_15	F: TGTCAGTCAAATTCTCGCAGG	(AAG)_11_	297–330	MN219560
	R: CGCTTTCGGCTTGTTGTAAG			
SP_Tri_10	F: TGAAACAAACAGTGGGCCG	(ATC)_9_	166–199	MN219559
	R: AAGAACCCGCCTGGTGAAG			

a Annealing temperature was 55°C for all loci.

b One microvariant allele was detected in each of these markers; these microvariants differ by 1 bp from the nearest (shorter) allele, and all other alleles derived from these microvariants differ by the expected length (according to the repeat motif).

c Alleles deviating by 1 bp from the expected allele size were observed in part of the individuals from the Hungarian population. These alleles are at least 9 bp longer than the last “regular” allele.

**Table 2:** Genetic properties of the 18 polymorphic microsatellite markers developed for *Salvia pratensis*.^a^

Locus	Czech Republic (*n* = 20)				Hungary (*n* = 19)				Poland (*n* = 19)				Germany (n = 20)			
	*A*	*H*_o_	*H*_e_	*f*	*A*	*H*_o_	*H*_e_	*f*	*A*	*H*_o_	*H*_e_	*f*	*A*	*H*_o_	*H*_e_	*f*
SP_Tri_36	9	0.75	0.79	0.05*	9	0.70	0.78	0.10*	4	0.70	0.64	–0.09	6	0.55	0.62	0.12
SP_Di_24	4	0.20	0.19	–0.07	4	0.55	0.44	–0.24	2	0.05	0.05	–0.03	4	0.50	0.44	–0.14
SP_Di_25	9	0.65	0.74	0.12*	9	0.90	0.80	–0.13*	7	0.40	0.65	0.38	7	0.80	0.77	–0.04
SP_Di_31	13	0.80	0.79	–0.01*	9	0.65	0.76	0.14	7	0.63	0.68	0.08	7	0.80	0.71	–0.13
SP_Di_27	6	0.65	0.63	–0.03*	6	0.30	0.42	0.29	5	0.20	0.23	0.13*	3	0.25	0.23	–0.11
SP_Tri_34	8	0.60	0.77	0.22*	7	0.55	0.64	0.15	6	0.84	0.70	–0.20	4	0.55	0.62	0.12
SP_Di_30	12	0.95	0.88	–0.08*	12	0.90	0.85	–0.06	15	0.95	0.89	–0.06	10	0.80	0.82	0.03*
SP_Di_02	7	0.56	0.50	–0.12*	8	0.74	0.78	0.06	13	0.85	0.84	–0.02*	5	0.70	0.71	0.01
SP_Di_06	5	0.35	0.60	0.42*	9	0.55	0.68	0.19*	4	0.85	0.62	–0.37	5	0.55	0.60	0.17
SP_Di_04	8	0.45	0.76	0.41*	13	0.53	0.86	0.39*	10	0.79	0.84	0.07*	9	0.70	0.71	0.03
SP_Di_08	10	0.79	0.79	0.00	9	0.73	0.76	0.04	11	0.89	0.83	–0.07*	6	0.55	0.48	0.09
SP_Tri_17	11	0.83	0.78	–0.07*	9	0.71	0.81	0.13*	13	0.61	0.85	0.28*	5	0.11	0.73	0.85*
SP_Tri_23	4	0.74	0.70	–0.05	6	0.56	0.60	0.07*	5	0.50	0.63	0.21	5	0.70	0.74	0.06
SP_Tri_04	4	0.29	0.26	–0.11	4	0.41	0.44	0.07	4	0.78	0.64	–0.22	3	0.45	0.37	–0.20
SP_Te_07	9	0.58	0.58	0.00	8	0.71	0.69	–0.03*	10	0.83	0.80	–0.04	7	0.35	0.63	0.44*
SP_Tri_06	6	0.58	0.53	–0.08	6	0.50	0.54	0.07	7	0.78	0.68	–0.14	4	0.35	0.53	0.34
SP_Tri_15	12	0.47	0.85	0.44*	13	0.80	0.89	0.10	9	0.69	0.82	0.16	14	0.65	0.79	0.17
SP_Tri_10	8	0.74	0.79	0.07	11	0.83	0.82	–0.02*	9	0.72	0.85	0.16	14	0.85	0.88	0.11
Overall	145	0.61	0.66	0.08*	152	0.65	0.70	0.08*	141	0.67	0.68	0.02*	118	0.57	0.64	0.11*

Note

*A* = number of alleles sampled; *f* = inbreeding coefficient; *H*_e_ = expected heterozygosity; *H*_o_ = observed heterozygosity; *n* = number of individuals sampled.

a The geographic origins of the populations analyzed are provided in Appendix 1.

* Significant deviation from Hardy–Weinberg equilibrium (*P* < 0.05).

We further cross‐amplified the newly developed markers in six other *Salvia* species (*S. aethiopis*,* S. austriaca*,* S. glutinosa*,* S. nemorosa*,* S. nutans*, and *S. verticillata*), each represented by five individuals (Appendix 1). Markers yielding microsatellite‐like peaks in at least four individuals per species were considered to successfully cross‐amplify. The highest cross‐amplification rate was obtained for *S. nemorosa*, for which 15 markers amplified (Table 3), whereas cross‐amplification to the distantly related *S. glutinosa* (see Will and Claßen‐Bockhoff, 2017) failed almost completely (Table 3). For the remainder of the *Salvia* species examined, 9–13 primer pairs yielded successful amplifications (Table 3).

**Table 3:** Results of cross‐amplification of 18 polymorphic microsatellites developed for *Salvia pratensis* in six other *Salvia* species.^a^^,^ b

Locus	*Salvia aethiopis*	*Salvia austriaca*	*Salvia glutinosa*	*Salvia nemorosa*	*Salvia nutans*	*Salvia verticillata*
SP_Tri_36	153–159	162–172	—	162–165	162–165	—
SP_Di_24	201	193–204	—	190–199	190–198	201–205
SP_Di_25	314	302–319	—	285–287	293–299	—
SP_Di_31	231–250	—	—	269–288	253–270	262–275
SP_Di_27	349	345–363	—	311	330–342	355
SP_Tri_34	221	211–215	—	226–230	221–243	217–224
SP_Di_30	156	148–151	—	166–185	148–179	151–190
SP_Di_02	—	—	—	—	—	—
SP_Di_06	229–239	—	—	—	—	261–263
SP_Di_04	229–231	214	256	243–261	228–244	261–265
SP_Di_08	143	141	—	149–157	153–180	159–161
SP_Tri_17	—	—	—	—	—	—
SP_Tri_23	231	—	—	207–226	—	—
SP_Tri_04	183	183	—	184	183–193	—
SP_Te_07	—	—	—	140–149	—	—
SP_Tri_06	—	—	—	146	148–163	151
SP_Tri_15	—	266–295	—	278–300	283–312	—
SP_Tri_10	—	—	—	158–189	156–179	—

Note

— = unsuccessful amplification.

a The geographic origins of the populations analyzed are provided in Appendix 1.

b Five individuals from each of these taxa were used for the cross‐amplification experiments. For each marker, the amplified fragment lengths are given in base pairs.

## CONCLUSIONS

We have developed 18 polymorphic microsatellite markers for *S. pratensis*, a perennial plant species representing one of the key taxa of European dry grasslands. In the near future, we will use these markers to reconstruct the phylogeography of this species in Europe and contribute to the reconstruction of the post‐glacial history of European dry grasslands, which are threatened habitats of high conservation value. Furthermore, at least nine of these markers were successfully cross‐amplified in five other *Salvia* species, providing resources for further genetic studies in the genus.

## AUTHOR CONTRIBUTIONS

K.K., P.V., and B.M. conceived and designed the study. All authors collected the plant material. K.K. supervised the laboratory work. K.K., P.V., and J.D. analyzed the data. K.K. and B.M. drafted the manuscript. All authors reviewed the manuscript and approved its final version.

## Data Availability

Sequences of the developed microsatellite loci were submitted to the National Center for Biotechnology Information (NCBI; accession numbers MN219547–MN219564). Illumina data have been deposited to the NCBI Sequence Read Archive (BioProject PRJNA564705, BioSample accession number SAMN12721148 [Illumina sequencing], and SRR10092015 [raw Illumina reads]).

## APPENDIX 1 Voucher and geographic information for *Salvia* samples used in this study.

**Table:**

Species	Voucher no.^a^	Location	Latitude	Longitude	*N*
*S. pratensis* L.	BRNU 665929	Czech Republic, Louny	50.41022	13.807042	20
	BRNU 667140	Hungary, Dorog	47.75062	18.74198	19
	BRNU 667298	Poland, Raclawice	50.33827	20.23332	19
	BRNU 667319	Germany, Könnern	51.67254	11.74234	20
*S. aethiopis* L.	BRNU 1503053	Bulgaria, Pazardzhik	42.11396	24.38720	5
*S. austriaca* Jacq.	BRNU 1503052	Bulgaria, Pazardzhik	42.11396	24.38720	5
*S. glutinosa* L.	BRNU 1502854	Iran, Fuman	37.17198	48.97948	5
*S. nemorosa* L.	BRNU 1502896	Poland, Skalbmierz	50.31159	20.40273	5
*S. nutans* L.	BRNU 1503050	Bulgaria, Pazardzhik	42.32028	24.39677	5
*S. verticillata* L.	BRNU 1503077	Bulgaria, Vratsa	43.19168	23.49425	5

Note

*N* = number of individuals.

^a^All herbarium specimen vouchers are stored in the herbarium of the Department of Botany and Zoology, Masaryk University, Brno, Czech Republic (BRNU).

## References

[aps311316-bib-0001] Belyayev, A. , J. Josefiová , M. Jandová , R. Kalendar , K. Krak , and B. Mandák . 2019 Natural history of a satellite DNA family: From the ancestral genome component to species‐specific sequences, concerted and non‐concerted evolution. International Journal of Molecular Sciences 20: 1201. 10.3390/ijms20051201 PMC6429384 30857296

[aps311316-bib-0002] Bolger, A. M. , M. Lohse , and B. Usadel . 2014 Trimmomatic: A flexible trimmer for Illumina sequence data. Bioinformatics 30: 2114–2120. 24695404 10.1093/bioinformatics/btu170 PMC4103590

[aps311316-bib-0003] Faircloth, B. C. 2008 MSATCOMMANDER: Detection of microsatellite repeat arrays and automated, locus‐specific primer design. Molecular Ecology Resources 8: 92–94. 21585724 10.1111/j.1471-8286.2007.01884.x

[aps311316-bib-0004] Habel, J. C. , J. Dengler , M. Janišová , P. Török , C. Wellstein , and M. Wiezik . 2013 European grassland ecosystems: Threatened hotspots of biodiversity. Biodiversity and Conservation 22: 2131–2138.

[aps311316-bib-0005] Kaplan, Z. , P. Koutecký , J. Danihelka , K. Šumberová , M. Ducháček , J. Štěpánková , L. Ekrt , et al. 2018 Distributions of vascular plants in the Czech Republic. Part 6. Preslia 90: 235–346.

[aps311316-bib-0006] Keenan, K. , P. McGinnity , T. F. Cross , W. W. Crozier , and P. A. Prodöhl . 2013 diveRsity: An R package for the estimation and exploration of population genetics parameters and their associated errors. Methods in Ecology and Evolution 4: 782–788.

[aps311316-bib-0007] Langmead, B. , and S. L. Salzberg . 2012 Fast gapped‐read alignment with Bowtie 2. Nature Methods 9: 357–359. 22388286 10.1038/nmeth.1923 PMC3322381

[aps311316-bib-0008] Miller, M. P. , B. J. Knaus , T. D. Mullins , and S. M. Haig . 2013 SSR_pipeline: A bioinformatic infrastructure for identifying microsatellites from paired‐end Illumina high‐throughput DNA sequencing data. Journal of Heredity 104: 881–885. 24052535 10.1093/jhered/est056

[aps311316-bib-0009] Molecular Ecology Resources Primer Development Consortium , J. An , Å. Bechet , Å. Berggren , S. K. Brown , M. W. Bruford , Q. Cai , A. Cassel‐Lundhagen , et al. 2010 Permanent genetic resources added to molecular ecology resources database 1 October 2009 – 30 November 2009. Molecular Ecology Resources 10: 404–408. 21565039 10.1111/j.1755-0998.2009.02827.x

[aps311316-bib-0010] Qian, J. , J. Song , H. Gao , Y. Zhu , J. Xu , X. Pang , H. Yao , et al. 2013 The complete chloroplast genome sequence of the medicinal plant *Salvia miltiorrhiza* . PLoS ONE 8: e57607. 23460883 10.1371/journal.pone.0057607 PMC3584094

[aps311316-bib-0011] Radosavljević, I. , J. Jakse , B. Javornik , Z. Satovic , and Z. Liber . 2011 New microsatellite markers for *Salvia officinalis* (Lamiaceae) and cross‐amplification in closely related species. American Journal of Botany e316–e318. 22003176 10.3732/ajb.1000462

[aps311316-bib-0012] Radosavljević, I. , Z. Satovic , J. Jakse , B. Javornik , D. Greguraš , M. Jug‐Dujaković , and Z. Liber . 2012 Development of new microsatellite markers for *Salvia officinalis* L. and its potential use in conservation‐genetic studies of narrow endemic *Salvia brachyodon* Vandas. International Journal of Molecular Sciences 13: 12082–12093. 23109901 10.3390/ijms130912082 PMC3472793

[aps311316-bib-0013] Schuelke, M. 2000 An economic method for the fluorescent labelling of PCR fragments. Nature Biotechnology 18: 233–234. 10.1038/72708 10657137

[aps311316-bib-0014] Van Loon, J. C. 1980 Reports *In* Löve A. [ed.], IOPB Chromosome number reports LXIX. *Taxon* 29: 718–720.

[aps311316-bib-0015] Will, M. , and R. Claßen‐Bockhoff . 2017 Time to split *Salvia* s.l. (Lamiaceae): New insights from Old World *Salvia* phylogeny. Molecular Phylogenetics and Evolution 109: 33–58. 28057553 10.1016/j.ympev.2016.12.041

